# Determinants of school-based sexuality education implementation in South Africa and Sweden: a comparative critical policy and evidence synthesis

**DOI:** 10.3389/fsoc.2026.1900545

**Published:** 2026-08-18

**Authors:** Ikekhwa Albert Ikhile, Mats Lundström, Azwihangwisi Mavhandu-Mudzusi, Camilla Palm, Ayobami Precious Adekola

**Affiliations:** 1 Department of Gender and Sexuality Studies, College of Human Sciences, University of South Africa, Pretoria, South Africa; 2 Department of Science, Mathematics and Society, Malmö University, Malmö, Sweden; 3 College of Human Sciences, University of South Africa, Pretoria, South Africa; 4 Department of Social Work, Malmö University, Malmö, Sweden

**Keywords:** comprehensive sexuality education, adolescent sexual and reproductive health, policy implementation, implementation fragility, implementation drift, marginalised learners, South Africa, Sweden

## Abstract

Policy commitment to school-based sexuality education does not guarantee inclusive classroom enactment. This comparative critical policy and evidence synthesis examines how sexuality education commitments are implemented in South Africa and Sweden, two rights-oriented but structurally different systems. South Africa formally locates comprehensive sexuality education (CSE) within Life Skills and Life Orientation and has developed national HIV, STI, and TB policy commitments and scripted lesson plans to support implementation. Sweden has a longer history of compulsory school sexuality education and, following curricular amendments implemented in 2022, uses the policy terminology sexuality, consent and relationships (SCR), with responsibility distributed across subjects and school actors. Guided by critical interpretive synthesis, policy triangle analysis and implementation scholarship, we conducted a structured identification and verification process across official policy repositories, monitoring reports, Swepub, Libris, Libsearch, publisher records, PubMed/PMC, Google Scholar and DOI records. Evidence was eligible when it addressed school-based policy content, educator practice, learner experience, inclusion of marginalised learners, teacher education, curriculum resources, inspection/monitoring or implementation support in either country. The synthesis indicates that South Africa and Sweden share an implementation problem but not the same implementation mechanism. South Africa illustrates implementation fragility: a legitimate CSE mandate is repeatedly destabilised before inclusive enactment becomes routine through uneven educator preparation, moral contestation, resource constraints, school-level variability and selective silencing of sexual and gender diversity. Sweden illustrates implementation drift: an institutionalised SCR architecture may lose equity depth through subject-dispersed responsibility, assumptions of progressiveness, variable teacher education, uneven learner participation and persistent silences around culture, religion, racism, disability, intersex, and asexuality. The concepts of fragility and drift extend existing language of implementation gaps by identifying distinct mechanisms through which policy commitments become weakened. Both contexts require sustained teacher education, stronger school-level coordination, meaningful youth participation, marginalised-learner monitoring, inclusive-resource audits and accountability indicators that assess what learners actually experience in classrooms.

## 1 Introduction

Comprehensive sexuality education (CSE) is an evidence-informed educational approach intended to equip children and young people with age-appropriate, scientifically accurate and rights-based knowledge concerning bodies, puberty, consent, relationships, gender equality, sexual and reproductive health and rights (SRHR), violence prevention, help-seeking and wellbeing. International guidance frames CSE not simply as health information but as a curriculum, pedagogical and rights-based commitment to dignity, safety, agency and inclusion (UNESCO et al., 2018; World Health Organization, 2026). Its implementation is therefore simultaneously an educational, public-health and social-justice concern.

Formal adoption, however, is not equivalent to equitable enactment. Sexuality education enters classrooms through curriculum language, educator confidence, teacher education, school leadership, curricular resources, learner participation, parental and community responses, inspection systems and wider political debates over gender, sexuality, childhood, religion and culture. A policy may be strong on paper while classroom delivery remains inconsistent, biomedical, moralising, heteronormative, ableist or silent on sexual and gender diversity. The implementation question is therefore not simply whether sexuality education exists, but which knowledge is taught, whose bodies and relationships are recognised, and which learners are protected or marginalised by everyday classroom practice (Durlak and DuPre, 2008; Lipsky, 1980; UNESCO et al., 2018).

South Africa and Sweden provide a theoretically productive comparison because both have formal commitments to equality, SRHR and school-based sexuality education, while their policy architectures and implementation conditions differ substantially. South Africa locates CSE within Life Skills and Life Orientation and has developed national HIV, STI and TB policy commitments and scripted lesson plans intended to support consistency, HIV/STI prevention, gender equality, safety and inclusive school environments (Department of Basic Education, 2017; Department of Basic Education, 2019). Yet empirical evidence repeatedly identifies teacher discomfort, moral and religious contestation, uneven school support, risk-focused implementation, resource constraints, limited learner voice and incomplete inclusion of disability and sexual and gender diversity (Adekola, 2024; DePalma and Francis, 2014; Hanass-Hancock et al., 2018; Mayeza and Vincent, 2019; Shefer and Macleod, 2015).

Sweden has a longer policy history: compulsory school sexuality education was introduced in 1955, and the 2022 curriculum reform renamed the field sexuality, consent and relationships (SCR), distributing responsibility across multiple school subjects and teachers (Skolverket, 2024). Swedish policy is strongly linked to gender equality, norm-conscious pedagogy, democratic values, student wellbeing and SRHR. Nonetheless, inspection evidence and recent scholarship show uneven quality, weak progression, insufficient student participation, variable teacher preparedness and persistent exclusions within an ostensibly progressive system (Andersson et al., 2020; Bengtsson and Bolander, 2020; Fingalsson, 2025; Junkala, 2023; Skolinspektionen, 2018, 2025).

This article argues that the comparative problem should not be framed as a simple contrast between a less established Global South system and a successful Global North model. Both countries demonstrate the gap between policy legitimacy and classroom equity, but the mechanisms of vulnerability differ. We conceptualise South Africa primarily as a case of implementation fragility, where a formal mandate remains vulnerable to destabilisation before equitable enactment becomes routine. We conceptualise Sweden primarily as a case of implementation drift, where long-institutionalised provision can lose its equity intent through routinisation, diffuse responsibility, local discretion or unexamined assumptions of inclusivity. These are analytical constructs, not fixed national labels; fragility can occur in Swedish schools, and drift can occur in South African schools where programmes become routine but lose responsive purpose.

The objectives of the synthesis are to (1) identify institutional, sociocultural, pedagogical, and governance determinants shaping school-based sexuality education implementation in South Africa and Sweden; (2) compare how inclusion and equity are enabled or constrained for marginalised learners; (3) explain how implementation fragility and implementation drift emerged from the comparative evidence; and (4) develop practical and research implications for policy, teacher education, youth participation and accountability.

## 2 Methods

### 2.1 Design and epistemological positioning

This article uses a comparative critical policy and evidence synthesis. It does not present an exhaustive scoping review or an intervention-effect review. This design was selected because the research question concerns how policy content, actors, institutional processes and sociocultural contexts interact to shape implementation. Such questions require interpretive integration of heterogeneous evidence, including official policy documents, inspection reports, teacher-education mappings, qualitative studies, learner-focused research, disability-focused scholarship, conceptual literature, and doctoral theses (Dixon-Woods et al., 2006; Grant and Booth, 2009).

Critical interpretive synthesis is appropriate when evidence is diverse, and the purpose is to construct an explanatory account rather than to aggregate effect sizes (Dixon-Woods et al., 2006). The analysis was sensitised by the Health Policy Triangle, which directs attention to policy content, context, actors, and processes (Walt and Gilson, 1994). Implementation scholarship further informed attention to fidelity, adaptation, capacity, acceptability, and sustainability (Durlak and DuPre, 2008). These frameworks were used as interpretive lenses, not as rigid coding templates.

### 2.2 Comparative case-selection rationale

The comparison follows a most-different, theoretically informed case logic. South Africa and Sweden differ in welfare-state history, schooling infrastructure, policy institutionalisation, resource distribution, monitoring capacity, and political–cultural debates around gender and sexuality. Yet both articulate rights-oriented sexuality education commitments and both seek to translate those commitments into school settings. The cases therefore allow examination of how different policy architectures encounter different implementation risks.

South Africa was selected because it combines strong constitutional commitments to equality with high SRHR need, HIV prevention imperatives, a formal school-based CSE mandate and continuing social contestation around sexuality, gender and sexual diversity. Sweden was selected because it has one of the longest histories of mandatory sexuality education, a strengthened SCR curriculum, a visible inspection infrastructure and extensive policy language around gender equality and norm-conscious education. The comparison is not designed to rank countries or to transfer one model wholesale to the other. It is designed to identify mechanism-level vulnerabilities that may be obscured when policy adoption is treated as implementation success (Meyer and Rowan, 1977; Walt and Gilson, 1994).

**Figure 1:**
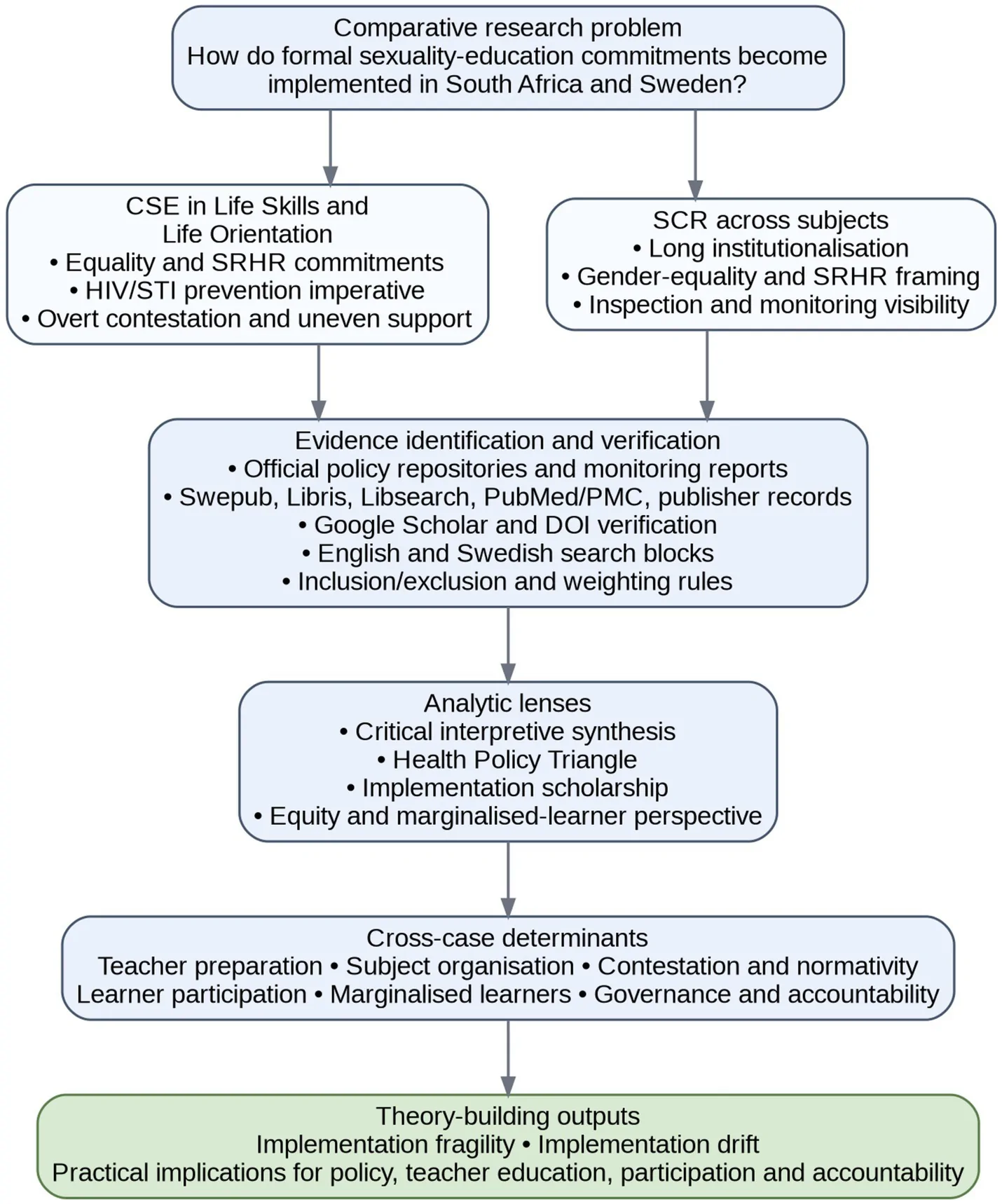
Comparative critical policy and evidence synthesis framework.

To orient the reader to the overall design of the study, Figure 1 presents the comparative critical policy and evidence synthesis framework that linked case selection, evidence identification, analytic lenses, and theory-building.

As shown in Figure 1, the synthesis moved iteratively from the comparative research problem and country contexts to verified evidence sources, analytic interpretation, and cross-case determinant mapping. This sequencing helped the manuscript remain transparent and reproducible while retaining the purpose of a comparative critical policy-and-evidence synthesis rather than claiming exhaustive systematic-review coverage.

### 2.3 Terminology and scope

CSE is used as the international comparative term for comprehensive, rights-based, age-appropriate and evidence-informed sexuality education (UNESCO et al., 2018; World Health Organization, 2026). In the South African sections, CSE refers to sexuality education embedded in Life Skills and Life Orientation and supported by DBE policy and scripted lesson plans (Department of Basic Education, 2017; Department of Basic Education, 2019). In the Swedish sections, SCR refers to the official policy terminology sexuality, consent, and relationships after the 2022 curriculum revision (Skolverket, 2024).

The unit of comparison was national policy implementation in school settings. Evidence was eligible when it addressed policy design, curriculum, teacher preparation, classroom delivery, learner experience, marginalised learner inclusion, curriculum resources, school leadership, inspection, monitoring, or intersectoral support. Documents focused solely on clinical SRHR services without a school-implementation link were excluded.

### 2.4 Evidence identification, screening, and verification

A structured search and bibliographic verification process was completed in June 2026. The search prioritised official national policy and monitoring sources, supplemented by empirical and conceptual scholarship directly relevant to school-based implementation. South African sources were identified through DBE/Gov.za, UNESCO Health and Education Resource Centre, PubMed/PMC, publisher records, Google Scholar and DOI records. Swedish sources were identified through Skolverket, Skolinspektionen, the Public Health Agency of Sweden, Swepub, Libris, Libsearch, DiVA records where institutionally relevant, publisher records, Google Scholar, and DOI records.

Search strings combined English and Swedish terms. English terms included the following: comprehensive sexuality education, school-based sexuality education, Life Orientation, Life Skills, sexuality diversity, learners with disabilities, teacher preparedness, implementation, learner participation, South Africa, Sweden, sexuality consent relationships, norm-critical, migration, and intellectual disability. Swedish terms included the following: sexualundervisning, sex- och samlevnadsundervisning, sexualitet samtycke relationer, skola, undervisning, lärare, elevperspektiv, funktionsnedsättning, intellektuell funktionsnedsättning, migration, nyanlända, normkritik, and ämnesintegrerad. Including Swedish-language search terms and Swedish discovery platforms strengthened reproducibility and reduced the risk of relying only on English-language scholarship.

Table 1 summarises the evidence identification, screening, and weighting protocol used to strengthen methodological transparency. It shows how source domains, date limits, eligibility criteria, exclusion rules, evidence weighting and synthesis logic were operationalised before interpretation.

**Table 1:** Evidence identification, screening, and weighting protocol.

Component	Operational approach	Eligibility/weighting decision	Purpose
Source domains	Official policy repositories; monitoring/inspection reports; Swepub, Libris, Libsearch, publisher records, Google Scholar, PubMed/PMC, and DOI records.	Official policy and inspection reports were treated as highly relevant to policy content and governance; empirical studies were weighted for classroom enactment and learner/teacher experience.	Improves transparency and avoids dependence on one database.
Date range	January 2000 to June 2026; earlier sources retained only for historical or conceptual grounding.	Priority was given to sources reflecting the South African CSE mandate, DBE SLPs, Swedish 2022 SCR reform, and post-reform evidence.	Captures contemporary implementation conditions while retaining policy history.
Inclusion criteria	School-based sexuality education policy, curriculum, educator delivery, learner voice, marginalised learners, teacher education, resources, or monitoring.	Evidence had to address South Africa or Sweden and have a direct school-implementation link.	Ensures alignment with the research question.
Exclusion criteria	Clinical SRHR services without school link; opinion pieces without evidence base; duplicate records; sources without sufficient bibliographic traceability.	Commentary was used only where it clarified policy context and not as empirical evidence.	Protects credibility and prevents over-claiming.
Evidence weighting	Official documents for policy intent; inspection reports for system monitoring; peer-reviewed/doctoral evidence for enactment; learner studies for lived classroom experience.	Author-team South African sources were interpreted alongside independent and official evidence, not as stand-alone proof.	Addresses evidence imbalance and interpretation-bias concerns.
Synthesis logic	Thematic coding followed by cross-case interpretation of stabilising and destabilising determinants.	Concepts were retained only when they explained repeated patterns across evidence types.	Supports theory-building rather than descriptive listing.

As shown in Table 1, the synthesis deliberately separates policy-intent evidence from classroom-enactment evidence and learner-experience evidence. This distinction is important because the article does not treat the existence of a national policy or curriculum as proof of inclusive implementation.

### 2.5 Data extraction, reflexivity, and mitigation of interpretation bias

A structured extraction matrix captured country, source type, policy level, implementation domain, participants or target population, determinant identified, equity implication, and relevance to the constructs of fragility or drift. For South Africa, several included studies were authored or co-authored by members of the manuscript team. This was managed through five safeguards: (1) author-derived evidence was not treated as determinative; (2) claims were triangulated with independent South African scholarship and official DBE sources; (3) no major interpretive claim was based solely on author-team scholarship; (4) Swedish co-authors reviewed whether comparative interpretations overstated South African-Swedish contrasts; and (5) the limitations explicitly distinguish evidence availability from implementation reality.

For Sweden, the evidence base contained a larger proportion of official policy, inspection and Swedish-language scholarship. To reduce language and source bias, the synthesis used Swedish search terms, Swedish repositories and doctoral theses alongside peer-reviewed publications. Where a Swedish source documented policy aspirations rather than classroom practice, this was coded as policy-content evidence rather than enactment evidence. This distinction was essential to avoid mistaking a comprehensive policy architecture for consistent classroom quality.

Evidence quality and strength were assessed interpretively rather than through a formal risk-of-bias score because the corpus included policy documents, inspection reports, qualitative studies, teacher-education mappings, learner-perspective studies, textbook analyses and doctoral theses. Greater interpretive weight was given to sources that were methodologically explicit, directly linked to school implementation, clearly traceable through official or peer-reviewed records, and proximate to classroom practice or learner experience. Official policies were treated as strong evidence of policy intent but weaker evidence of enactment; inspection reports were treated as strong evidence of system-level quality concerns; peer-reviewed and doctoral empirical studies were treated as stronger evidence of classroom, teacher and learner experience; and conceptual or historical sources were used mainly to interpret policy trajectories and normative assumptions (Dixon-Woods et al., 2006; Grant and Booth, 2009). Cross-source convergence was used as the principal confidence criterion: claims supported by policy, inspection and empirical classroom evidence were treated as higher-confidence than claims resting on a single report, one institution or self-reported educator perceptions.

This approach means that the conclusions should be read as theoretically grounded and evidence-informed rather than statistically generalisable. The strongest claims in the manuscript are those supported across multiple evidence types, for example the recurring role of teacher preparedness, learner participation, marginalised-learner inclusion and governance. More cautious claims are used where evidence is concentrated in one source type, one country, or a small number of classroom studies. Accordingly, the synthesis distinguishes robust recurring determinants from provisional country-specific interpretations and avoids inferring national prevalence from small qualitative samples.

### 2.6 Analytic strategy and concept generation

The analysis proceeded in three stages. First, evidence was coded descriptively for curriculum mandate, educator preparation, subject organisation, contestation, learner participation, marginalised learner inclusion, resources, school leadership, monitoring, and intersectoral support. Second, codes were grouped into cross-country determinants, with attention to whether they stabilised implementation, destabilised implementation, or allowed inequity to persist despite formal provision. Third, the comparative constructs implementation fragility and implementation drift were developed to explain the different mechanisms through which formal commitments weakened in each setting.

Implementation fragility was generated from evidence showing that legitimate policy may remain vulnerable before equitable practice becomes routine. Implementation drift was generated from evidence showing that institutionalised provision can become uneven or insufficiently inclusive when responsibility is diffused, quality is assumed, or accountability fails to track marginalised learner experience. These constructs were compared against established concepts such as implementation gap, policy-practice divergence, implementation fidelity, and institutional decoupling (Durlak and DuPre, 2008; Meyer and Rowan, 1977; Walt and Gilson, 1994).

## 3 Results: comparative evidence synthesis

### 3.1 Policy architectures: mandate, institutionalisation, and risk of over-reading policy intent

South Africa has a formal policy mandate for school-based CSE through Life Skills and Life Orientation. The DBE HIV, STI and TB policy positions schools as protective environments in which learners, educators, and officials have rights to information, protection from stigma, and access to means of preventing HIV, STIs, TB, and unintended pregnancy (Department of Basic Education, 2017). DBE scripted lesson plans were developed to support more standardised delivery across grades and to address persistent concerns about teacher confidence and curriculum consistency (Department of Basic Education, 2019; Government of South Africa, 2019). The strength of this architecture is that it provides a visible mandate and teaching support. Its vulnerability is that teaching remains mediated by educator beliefs, local school capacity, community contestation, and uneven resources (Adekola, 2024; Koch and Wehmeyer, 2021; Mayeza and Vincent, 2019).

Sweden’s SCR architecture is more historically institutionalised. Compulsory sexuality education was introduced in 1955, and the 2022 reform strengthened the area by renaming it sexuality, consent and relationships and embedding it across subjects and school responsibilities (Skolverket, 2024). The Swedish model therefore appears structurally robust. However, inspection evidence has repeatedly shown uneven quality, insufficient continuity, variable student participation, and weak school-level coordination (Skolinspektionen, 2018, 2025). Recent Swedish curriculum-reform debate, including proposals to relocate SCR more explicitly from the introductory parts of curricula into subject syllabi, further illustrates that cross-curricular responsibility remains politically and administratively unsettled (Government Offices of Sweden, 2026; Swedish Parliament, 2026).

The comparison indicates that policy architecture should be read as an implementation condition, not as an implementation outcome. South Africa demonstrates that explicit policy and lesson materials can still be fragile when institutional support is uneven. Sweden demonstrates that long-standing institutionalisation can still drift when responsibility is distributed across subjects without sufficient training, coordination, monitoring, and learner-centred accountability (Andersson et al., 2020; Fingalsson, 2025; Junkala, 2023; Skolverket, 2024).

### 3.2 Educator preparation, teacher discretion, and subject logics

Educator capacity is a shared determinant across both contexts, although it operates through different mechanisms. In South Africa, studies describe teacher discomfort, moral judgement, limited training, uncertainty about how to teach sexuality diversity, and reliance on personal beliefs when policy and pedagogical support are unclear (Adekola, 2024; DePalma and Francis, 2014; Swanepoel, 2017). Learner-focused research further suggests that adolescents often experience Life Orientation sexuality education as judgemental, gendered, repetitive, or disconnected from their actual questions and lived contexts (Mayeza and Vincent, 2019).

In Sweden, educator-capacity concerns do not arise from policy absence but from subject-dispersed implementation, uneven teacher education and the demands of addressing sensitive or contested topics. Andersson et al. (2020) mapped sexuality and relationships education in Swedish teacher education and found considerable variation across universities and programmes. Swedish teachers may feel relatively confident teaching anatomy, reproduction, contraception, and STIs, but less confident addressing norms, LGBTQI+ issues, culture, religion, racism, identity, relationships, and dialogical pedagogy (Junkala, 2023; Planting-Bergloo, 2023; Skolinspektionen, 2018).

The Swedish evidence also shows that subject integration (ämnesintegrering) is not automatically comprehensive. Junkala’s work on biology teaching and textbooks shows that biological subject logics can privilege medical, reproductive and STI-focused content, while leaving disability, intersex, asexuality, and broader sociocultural questions under-addressed (Junkala, 2023; Junkala et al., 2022). Planting-Bergloo (2023) similarly shows that natural-science sexuality education and interdisciplinary teaching produce different enactments; biological and medical content should not be separated from history, culture, religion, societal norms, gender equality, communication, and consent. The determinant is therefore not only whether teachers are trained, but whether the school system gives teachers time, content knowledge and collaborative structures to translate SCR beyond a narrow subject logic.

In both countries, teacher discretion has double effects. It can enable contextual responsiveness when teachers are skilled, reflexive and supported. It can also narrow the curriculum when teachers avoid contested content, reproduce stereotypes or default to biomedical and risk-focused instruction. This finding supports the need for teacher education that combines content knowledge, inclusive pedagogy, classroom dialogue management, disclosure-sensitive practice and reflexive engagement with power (Andersson et al., 2020; Junkala, 2023; Macleod and du Plessis, 2024; Planting-Bergloo, 2023).

### 3.3 Contestation, normativity, and the changing location of “sensitive” knowledge

In South Africa, sexuality education is frequently shaped by overt moral, religious, and community contestation. CSE can become publicly contested as an intrusion into parental authority or as a threat to religious or cultural norms, especially when it includes sexual diversity, contraception, consent, pleasure, gender equality, or learner sexual agency (Francis and McEwen, 2024; Shefer and Macleod, 2015; Ubisi, 2023). Such contestation can lead to selective implementation: teachers may teach HIV and pregnancy prevention while avoiding sexual orientation, gender identity, pleasure, consent, or learner-centred dialogue (DePalma and Francis, 2014; Swanepoel, 2017).

Sweden is often framed as more progressive, but Swedish evidence shows that normativity has not disappeared; it has shifted form. LGBTQI+ perspectives are more visible in recent textbooks and policy language than in earlier periods, but they may still be taught through heteronormative assumptions or as add-ons rather than integral content (Bengtsson and Bolander, 2020; Junkala et al., 2022; Schindele et al., 2023). Junkala (2023) shows that intersex, asexuality, and disability can remain absent even when sexual orientation and transgender content are visible. Biström (2022) further demonstrates that lower-secondary textbooks may frame sexuality around reproduction, androcentrism, phallocentrism, and monogamy.

A major Swedish gap concerns culture, religion, and racism. Norm-critical pedagogy has been promoted as a strategy for inclusion, yet scholarship shows tensions when norm-critical approaches address LGBTQI+ inclusion while insufficiently recognising racialisation, religion, tradition, Swedish exceptionalism, and migrant learners’ respectability work (Bengtsson and Bolander, 2020; Bredström, 2005; Bredström et al., 2018; Fingalsson and Junkala, 2025). Arabic-speaking migrant parents in Sweden have also expressed concern about school sexuality education while still valuing knowledge when it is communicated in culturally and religiously responsive ways (Herzig van Wees et al., 2021).

The comparative implication is that equity can be undermined by both explicit resistance and implicit normalisation. South Africa’s vulnerability is often visible as overt contestation. Sweden’s vulnerability is often less visible because progressive policy language can mask persistent silences. This distinction supports the fragility/drift argument: resistance destabilises implementation before it becomes routine, while routinised progressiveness can drift away from the learners whose identities remain insufficiently recognised.

### 3.4 Learner participation, co-creation, and the risks of tokenistic voice

Learner participation emerged as a central determinant across both countries. In South Africa, learner-focused studies show that young people often want sexuality education that is relevant, non-judgemental, dialogical, and responsive to their social realities (Adekola and Mavhandu-Mudzusi, 2023; Mayeza and Vincent, 2019). Participatory approaches can improve relevance by allowing adolescents to co-create praxis and identify local barriers to safer sexual decision-making (Koch and Beyers, 2023; Matahela et al., 2025). However, learner participation requires skilled facilitation, because classrooms are not neutral spaces: gender hierarchies, homophobia, disability stigma, and peer ridicule can shape who speaks and who remains silent.

Swedish evidence similarly shows that students often report adequate information on anatomy, pregnancy, and condoms but ask for more on gender, relationships, norms, LGBTQI+ perspectives, HIV, pleasure, misconceptions, pornography, virginity, and the myth of the hymen (Planting-Bergloo, 2023; Unis and Sällström, 2020). The Swedish Schools Inspectorate found that many schools did not sufficiently involve students in planning, evaluation, or development of sexuality education (Skolinspektionen, 2018, 2025). Schindele et al. (2023) further show that young people in Sweden do not have equal perceived knowledge from school-based sexuality education and that LGBTQI+ youth face particular barriers in using such education for sexual health literacy.

The Swedish classroom literature complicates simple calls for co-production. Junkala (2023) shows that teachers sometimes avoid open dialogue to control the unexpected, manage discriminatory language or protect learners from discomfort. Yet the same work also shows that when teachers skilfully engage unexpected student comments, new learner-relevant content can emerge and student engagement can deepen. Hildebrand (2025) similarly cautions that student talk may reproduce stereotypes and normative assumptions if it is not actively facilitated. Learner voice should therefore be treated as a quality indicator only when paired with safeguarding, teacher skill, anti-discriminatory norms, and mechanisms for marginalised learners to participate without exposure or harm.

### 3.5 Marginalised learners: LGBTQI+, disability, migrants, and rurality

Because inclusion is a central implementation concern, this synthesis treats marginalised learner perspectives not as a general policy value but as an implementation test. For LGBTQI+ learners, South African evidence shows that sexuality diversity may be avoided, moralised, or misunderstood by Life Orientation teachers despite constitutional commitments to equality (DePalma and Francis, 2014; Swanepoel, 2017). Swedish evidence shows greater curricular visibility of LGBTQI+ perspectives, but continuing risk of heteronormativity, incomplete integration, and barriers to sexual health literacy among sexual-minority youth (Junkala et al., 2022; Lukkerz and Arvidsson, 2025; Schindele et al., 2023).

For learners with disabilities, South African research shows that educators may lack disability-inclusive content, accessible materials, and confidence to deliver CSE to learners with disabilities, thereby limiting these learners’ rights to information, safety, and sexual agency (Hanass-Hancock et al., 2018). Swedish scholarship on intellectual disability is more mixed. Löfgren-Mårtenson (2012) found that young people with intellectual disabilities experienced sex education that was often insufficient, heteronormative, and focused on love, friendship, risk, and protection. Nelson et al. (2020), however, found that teachers in special-needs schools could be motivated, prepared, and supported by resources to cover a broader SRHR range, while still finding culture and religion difficult. The Swedish Schools Inspectorate’s adapted-school review further underlines that inclusion requires explicit planning, student participation, and quality assurance (Skolinspektionen, 2025). The difference suggests that disability inclusion depends strongly on teacher confidence, institutional support, resources, and willingness to treat learners with disabilities as sexual rights-bearing subjects rather than merely vulnerable subjects.

For migrant learners and families, the Swedish evidence is particularly important. Bredström (2005), Bredström et al. (2018), and Fingalsson and Junkala (2025) show that Swedish sexuality education may construct Swedishness as progressive while positioning racialised or migrant others as culturally problematic. The RFSU report on young people with experience of honour norms found that some participants experienced sexuality education as initially shocking but later became curious, while also reporting that teaching could feel distant from their realities or make them feel singled out (RFSU, 2022). Herzig van Wees et al. (2021) show that migrant parents may be concerned about school sex education while wanting children to receive knowledge adapted to family values. These sources prevent simplistic framing of Sweden as uniformly inclusive and demonstrate that migrant inclusion requires cultural responsiveness without surrendering rights-based commitments.

For rural learners, South African evidence highlights spatial inequality, resource constraints, educator preparedness gaps, and community norms as determinants of implementation (Adekola, 2024; Adekola and Mavhandu-Mudzusi, 2023; Matahela et al., 2025). Rurality operates as more than geography: it shapes teacher availability, school support, confidentiality, local moral surveillance, referral pathways, and learner access to youth-friendly services. The comparative lesson is that marginalisation differs by context, but implementation equity must be assessed through the lived experience of learners whose sexualities, bodies, languages, cultures, disabilities, or locations are least likely to be recognised by routine teaching.

### 3.6 Governance, monitoring, and accountability

Governance determines whether sexuality education remains dependent on individual teacher motivation or becomes a supported institutional practice. South African implementation is strengthened by national policy and scripted lesson plans but remains constrained by variable school leadership, resource inequality, uneven teacher training, and dependence on external partners or NGOs in some settings (Department of Basic Education, 2017; Department of Basic Education, 2019; Koch and Wehmeyer, 2021; Pillay and Mfidi, 2024). Intersectoral collaboration can support referral and psychosocial response, but it requires clear role allocation, follow-up mechanisms and school-health linkages (Macleod and du Plessis, 2024; Pillay and Mfidi, 2024).

Sweden has stronger formal monitoring and a national SRHR framework connecting school education with wider public health and equality commitments (Public Health Agency of Sweden, 2023; Skolinspektionen, 2018, 2025). However, monitoring visibility can also create an evidence imbalance: Sweden’s implementation challenges may appear more visible because inspection systems document them, whereas South African evidence may appear fragmented because it is distributed across empirical studies and programme literature. The synthesis therefore avoids concluding that Sweden is more problematic merely because it has more inspection data; instead, it compares how each evidence system reveals different aspects of implementation.

Recent Swedish curriculum-reform debates reinforce the importance of accountability. If SCR responsibilities are re-specified through subject syllabi or narrowed in practice, explicit monitoring of school-wide coordination, teacher preparedness, learner participation and marginalised-learner inclusion becomes even more important (Government Offices of Sweden, 2026; Skolinspektionen, 2025; Swedish Parliament, 2026). This does not mean that cross-curricular policy language alone guarantees quality; rather, it means that any architecture—cross-curricular or subject-specific—requires named ownership, teacher education, curriculum resources, and enforceable equity indicators.

Table 2 maps the expanded evidence corpus used in the synthesis and clarifies the contribution of each evidence domain. It shows how policy documents, inspection reports, teacher-education evidence, learner studies, disability-focused scholarship, migration-related studies, and textbook analyses from both countries were integrated into the comparative interpretation.

**Table 2:** Expanded evidence corpus and contribution to the comparative synthesis.

Country	Evidence domain	Representative sources	Contribution to synthesis
South Africa	Policy mandate and materials	Department of Basic Education (2017); Department of Basic Education (2019)	Formal CSE mandate, HIV/STI/TB prevention, safe-school responsibilities, and scripted lesson support.
South Africa	Teacher enactment and contestation	DePalma and Francis (2014); Swanepoel (2017); Adekola (2024); Ubisi (2023)	Teacher beliefs, moral discomfort, sexuality diversity avoidance, and contested implementation.
South Africa	Learner experience and co-creation	Mayeza and Vincent (2019); Adekola and Mavhandu-Mudzusi (2023); Koch and Beyers (2023)	Need for relevant, dialogical, and youth-responsive teaching.
South Africa	Disability, rurality, and support systems	Hanass-Hancock et al. (2018); Matahela et al. (2025); Macleod and du Plessis (2024); Pillay and Mfidi (2024)	Disability inclusion, rural constraints, referral capacity, and intersectoral collaboration.
Sweden	Policy and monitoring	Skolverket (2024); Skolinspektionen (2018, 2025); Public Health Agency of Sweden (2023)	SCR terminology, cross-curricular responsibility, uneven quality, learner participation, and adapted-school monitoring.
Sweden	Teacher education and subject logics	Andersson et al. (2020); Junkala (2023); Planting-Bergloo (2023)	Variation in teacher preparation; biology/natural-science logics may medicalise content; interdisciplinary enactment broadens perspectives.
Sweden	Norm-critical pedagogy, migration, and racialisation	Bengtsson and Bolander (2020); Bredström et al. (2018); Bredström (2005); Herzig van Wees et al. (2021); RFSU (2022)	Tensions around LGBTQI+ inclusion, colour-blindness, religion, culture, racism, and migrant-family concerns.
Sweden	Textbooks, disability, and learner perspectives	Junkala et al. (2022); Biström (2022); Löfgren-Mårtenson (2012); Nelson et al. (2020); Schindele et al. (2023)	Visibility and silences in materials; intellectual-disability inclusion; unequal perceived knowledge among young people.

The evidence distribution in Table 2 confirms that the two countries are not supported by identical types of evidence. South African evidence is especially strong on local implementation barriers and learner/educator experiences, while Swedish evidence is strengthened by inspection reports, curriculum documentation, teacher-education mapping and Swedish-language doctoral research. Building on this evidence map, Table 3 distils the main determinants into a cross-country implementation matrix.

**Table 3:** Determinants of school-based sexuality education implementation in South Africa and Sweden.

Determinant	South Africa: dominant vulnerability	Sweden: dominant vulnerability	Comparative implication
Policy architecture	Formal mandate and SLPs exist, but implementation remains uneven across schools and communities.	Long-standing SCR mandate exists, but responsibility may be diffused across subjects and school actors.	Policy legitimacy is necessary but insufficient for classroom equity.
Teacher preparation	Uneven training, discomfort, limited resources and reliance on personal beliefs.	Variable teacher education; confidence stronger for anatomy/STIs than for norms, religion, culture, LGBTQI+ issues and dialogue.	Teacher education must address content, pedagogy, power, safeguarding, and reflexivity.
Subject organisation	CSE embedded in Life Skills/Life Orientation, but may narrow to HIV, pregnancy and risk.	Subject-integrated SCR may broaden perspectives but may also medicalise content when dominated by Biology/Natural Science.	Implementation depends on how subject logics organise what is teachable.
Contestation and normativity	Overt moral and religious opposition can narrow or silence content.	Progressive narratives can obscure colour-blindness, heteronormativity and silences around intersex, asexuality, disability, and racism.	Equity can be undermined by both resistance and routinised normalisation.
Learner participation	Learners call for relevant, non-judgemental and co-created pedagogy.	Inspections and studies show insufficient learner participation and uneven perceived knowledge.	Learner participation should be a monitored quality indicator.
Marginalised learners	LGBTQI+ learners, learners with disabilities and rural learners face invisibility and access barriers.	LGBTQI+ perspectives are more visible, but disability, migrant, intersex, asexuality, and racialised perspectives remain uneven.	Inclusion must be measured through marginalised learner experience, not only policy language.
Governance and accountability	Variable school leadership, referral capacity, and intersectoral support.	Stronger monitoring, but risk of drift where local responsibility is unclear or assumed.	Systems require named ownership, routine audits and equity-sensitive accountability.

### 3.7 Cross-country determinant matrix

Table 3 presents the determinant matrix derived from the cross-case synthesis. It compares the dominant South African and Swedish vulnerabilities under each determinant and identifies the comparative implication for implementation theory and practice.

Table 3 shows that implementation weakness cannot be reduced to a single policy-practice gap. Instead, similar determinants operate through different mechanisms: South Africa is more exposed to fragility before routine enactment is secured, while Sweden is more exposed to drift within an already institutionalised architecture. The discussion described later develops these mechanism-level implications.

## 4 Discussion

### 4.1 Principal comparative contribution

This synthesis contributes a comparative implementation account rather than a descriptive country comparison. The findings indicate that sexuality education policy can weaken through different pathways. In South Africa, formal CSE legitimacy is repeatedly destabilised by capacity gaps, moral contestation, school-level variability and resource constraints. In Sweden, institutionalised SCR can lose equity depth through subject-dispersed responsibility, assumed progressiveness, uneven teacher preparation, insufficient learner participation, and unmonitored silences. These patterns are named implementation fragility and implementation drift.

The distinction is important because generic references to an implementation gap can conceal the mechanism that requires intervention. A South African implementation problem may require stronger teacher support, school leadership, community engagement, and protection against the selective silencing of contested content. A Swedish implementation problem may require preventing routinised provision from becoming uneven, biomedical, colour-blind, or insufficiently accountable to marginalised learners. Both require stronger teacher education and monitoring, but the policy levers differ.

Across the two cases, convergence is strongest around four determinants: uneven teacher preparation, the narrowing effects of subject logics, insufficient learner participation, and the incomplete recognition of marginalised learners. Divergence lies in how these determinants are produced. In South Africa, they are more often intensified by resource inequality, overt moral contestation, and variable school support; in Sweden, they are more often sustained by dispersed responsibility, institutional complacency, and the assumption that progressive policy language already guarantees inclusion. These contextual differences explain why similar classroom outcomes—biomedical narrowing, selective silence, and unequal learner recognition—arise through different implementation pathways.

### 4.2 Positioning implementation fragility and implementation drift against existing concepts

Implementation fragility refers to a condition in which a legitimate policy mandate exists but has not yet become sufficiently routinised, resourced and protected to withstand contestation or institutional unevenness. It differs from implementation gap because it specifies a pre-routinisation vulnerability: policy is not merely failing to reach practice; it is repeatedly destabilised before inclusive practice becomes normalised. It differs from fidelity because the issue is not only adherence to a planned programme but whether the conditions exist for a rights-based curriculum to survive local resistance and resource scarcity (Durlak and DuPre, 2008).

Implementation drift refers to a condition in which a policy area is institutionalised but gradually departs from its equity promise through routinisation, diffuse responsibility, local discretion, or weak accountability. It differs from policy-practice divergence because it highlights weakening within an apparently established system. It overlaps with institutional decoupling, where formal structures become disconnected from practice, but adds an equity-specific focus on the erosion of inclusion for marginalised learners (Meyer and Rowan, 1977). In the Swedish case, drift does not mean absence of sexuality education; it means that provision may continue while learner participation, cultural responsiveness, disability inclusion or recognition of less visible identities weakens.

The constructs should not be read as national stereotypes. They are portable mechanisms. A Swedish school may experience fragility where teachers are newly required to teach SCR without preparation. A South African school may experience drift where CSE is institutionalised but becomes repetitive, risk-only or disconnected from learner realities. The conceptual value lies in identifying the mechanism to be monitored and corrected.

**Figure 2:**
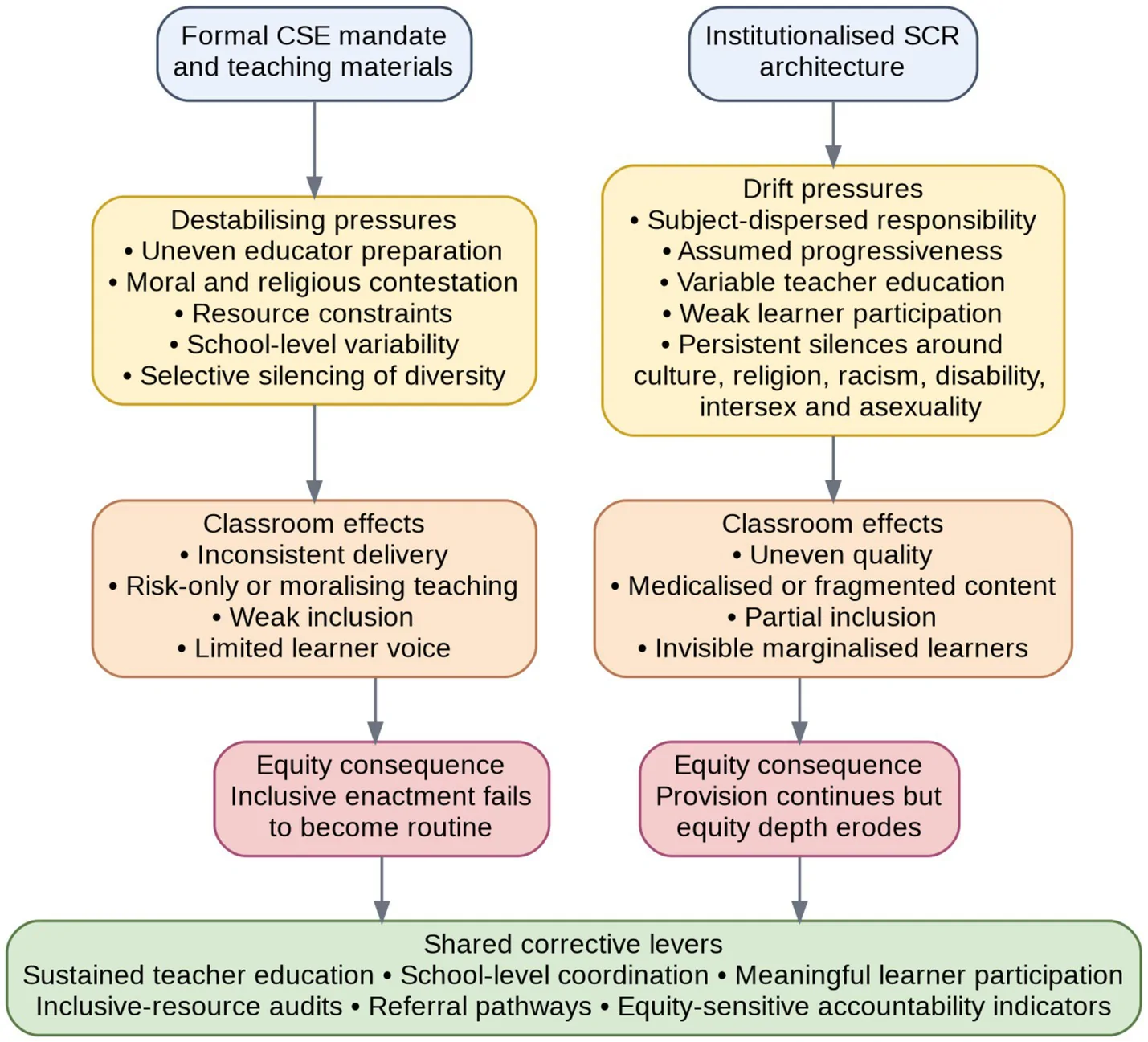
Implementation under contestation: Pathways of implementation fragility and implementation drift.

Figure 2 translates the manuscript’s core conceptual contribution into two implementation pathways and shows how sexuality-education commitments may weaken through either destabilisation before routinisation or erosion after institutionalisation.

As Figure 2 indicates, the contrast is not between the presence and absence of sexuality education, but between different mechanisms through which equity can be undermined. The figure also highlights the shared corrective levers required across both contexts, namely sustained teacher education, stronger school-level coordination, meaningful learner participation, inclusive-resource audits, referral pathways, and equity-sensitive accountability indicators.

### 4.3 Critical inconsistencies and unresolved issues in the evidence

The evidence contains several tensions. First, standardisation can improve consistency but also constrain responsiveness. South African scripted lesson plans can help teachers cover difficult content, yet they do not automatically produce dialogical, context-responsive or inclusive pedagogy (Department of Basic Education, 2019; Koch and Wehmeyer, 2021). Sweden’s subject-integrated model can broaden sexuality education across disciplinary perspectives, yet it may also diffuse ownership and reproduce subject-specific silences (Junkala, 2023; Planting-Bergloo, 2023).

To make the critical synthesis more explicit, Table 4 separates areas of convergence from areas of divergence and identifies contextual explanations for apparently conflicting findings across the South African and Swedish evidence bases.

**Table 4:** Convergence, divergence, and contextual explanations in the South Africa–Sweden synthesis.

Analytic area	Convergence across both countries	Divergence, conflicting evidence and contextual explanation
Policy commitment versus classroom enactment	Both countries have formal sexuality-education commitments and rights-oriented policy language, yet neither policy architecture guarantees equitable classroom practice.	South Africa shows more visible fragility because policy is often exposed to moral contestation, resource constraints and school-level variability. Sweden shows more visible drift because long institutionalisation can mask uneven quality, diffuse ownership, and unexamined assumptions of progressiveness.
Teacher preparedness	In both systems, teachers require more than content knowledge; they need confidence, facilitation skills, safeguarding awareness, and reflexive capacity to address power, stigma, and disclosure.	South African studies emphasise discomfort, personal beliefs, and limited support in contested or under-resourced schools. Swedish studies show confidence with anatomy or STIs but less confidence with norms, culture, religion, racism, LGBTQI+ issues, and dialogical pedagogy.
Learner participation	Learners in both settings require relevant, non-judgemental and participatory education that recognises their lived realities.	Evidence supports co-creation but also cautions that classroom dialogue can reproduce stereotypes or expose marginalised learners if facilitation is weak. This creates a tension between participation as empowerment and participation as a potential site of harm.
Marginalised learners	LGBTQI+ learners, learners with disabilities, migrant learners, and rural learners test whether implementation is truly equitable rather than only formally inclusive.	Sweden has more visible LGBTQI+ representation in some resources but continuing silences around intersex, asexuality, disability, race, and migration. South Africa has strong constitutional equality language but persistent avoidance or moralisation of sexuality diversity in classroom practice.
Monitoring and evidence visibility	Both countries need accountability indicators that assess learner experience, teacher competence, and inclusive enactment rather than policy existence alone.	Sweden has stronger inspection visibility, which may make problems easier to document. South African evidence is more dispersed across empirical and programme studies. The apparent difference is therefore partly substantive and partly a product of unequal monitoring infrastructures.
Quality and strength of evidence	Evidence in both countries consistently identifies teacher preparedness, learner participation and inclusive governance as implementation determinants.	Swedish conclusions draw more heavily on inspection reports, curriculum mapping and doctoral studies, whereas South African conclusions draw more heavily on qualitative and programme-level evidence. This asymmetry increases confidence in recurring cross-case mechanisms but requires caution when comparing national prevalence or causal magnitude.

Table 4 clarifies that the comparative contribution lies not in treating one country as successful and the other as deficient, but in identifying different mechanisms through which similar policy aspirations become weakened. The table also shows why contradictory evidence should not be treated as a weakness of the review; rather, such contradictions reveal how implementation depends on local institutional histories, evidence systems, subject organisation, and sociocultural contestation.

Second, learner participation is essential but not risk-free. Participatory and co-created approaches can improve relevance and learner agency (Koch and Beyers, 2023; Matahela et al., 2025). However, classroom dialogue can reproduce stereotypes, expose marginalised learners or generate discriminatory talk if teachers lack facilitation skills (Hildebrand, 2025; Junkala, 2023). Meaningful participation therefore requires anti-discriminatory classroom norms, anonymous feedback channels, safeguarding protocols and teacher competence in handling disclosure, discomfort, and peer power.

Third, inclusion has become more visible but remains uneven. Swedish evidence shows expanded LGBTQI+ visibility in some resources, but disability, intersex, and asexuality remain sparse or absent in textbooks and classroom enactment (Junkala et al., 2022). South African evidence shows constitutional equality commitments but continuing teacher avoidance of sexuality diversity (DePalma and Francis, 2014; Swanepoel, 2017). The unresolved issue is how to move from symbolic inclusion to ordinary, non-tokenistic, and developmentally appropriate recognition across curriculum, teaching materials, and classroom interaction.

Fourth, monitoring can illuminate problems but also shape what counts as evidence. Sweden’s formal inspection infrastructure generates visible system-level findings. South African implementation evidence is more dependent on empirical research and programme studies. Comparative claims must therefore separate observed implementation patterns from differences in data infrastructure, because evidence imbalance may reflect source availability as well as real implementation differences.

The strength of the evidence also differs by claim. Confidence is highest for the recurring importance of teacher preparation, learner participation, and governance because these patterns appear across official reports, peer-reviewed studies, and doctoral research. Confidence is moderate for country-level differences in fragility and drift because the evidence architectures are asymmetrical and direct cross-national studies are scarce. Evidence is weakest for causal claims about which implementation strategy produces superior learner outcomes, as few longitudinal, observational, or intervention studies directly connect policy architecture to classroom practice and learner-reported outcomes.

### 4.4 Practical implications for policy and implementation

The practical value of the synthesis is strengthened when recommendations are allocated to the actors who can act on them. Table 5 therefore translates the findings into concrete actions for policymakers, curriculum developers, teacher educators, and school administrators.

**Table 5:** Concrete implementation recommendations for key education-system actors.

Actor group	Concrete recommendation	Suggested accountability indicator
Policymakers and education departments	Move beyond policy adoption by funding recurrent teacher training, school-level coordination, disability-accessible materials, referral pathways, and monitoring of marginalised-learner experience. Require each education district to publish a costed three-year implementation plan and disaggregate monitoring by school type, disability, location, and learner group.	Annual reporting on teacher training coverage, school implementation plans, referral linkages and learner-reported safety, inclusion and relevance. Include annual targets, responsible units, budget lines, corrective actions, and public reporting of implementation gaps.
Curriculum developers and resource designers	Audit curricula, scripted lessons, textbooks and digital resources for heteronormativity, ableism, racialised assumptions and silences around intersex, asexuality, disability, migration, culture and religion. Establish a 2-year revision cycle using learner panels, disability-accessibility review and intersectional content standards.	Documented inclusive-resource audit with revision dates, stakeholder input, and evidence that marginalised identities are integrated rather than treated as add-ons. Record which identified omissions were corrected and whether revised materials were piloted with marginalised learners.
Teacher educators and professional-development providers	Prepare teachers to facilitate difficult knowledge, including consent, pleasure, pornography, sexual diversity, disability, racism, migration, religion, disclosure, distress, and discriminatory classroom talk. Require assessed micro-teaching, scenario-based facilitation and supervised practicum rather than attendance-only training.	Pre-service and in-service modules with assessed competence in content knowledge, dialogical pedagogy, safeguarding, anti-discrimination, and referral practice. Report demonstrated competence before certification and follow-up observation of classroom enactment within 6–12 months.
School administrators and principals	Assign named responsibility for sexuality education coordination, protect teachers who implement rights-based content, create anonymous learner-feedback mechanisms, and connect classrooms to school-health and psychosocial support. Conduct termly coordination meetings, protect curriculum time and review anonymous learner feedback at least twice per year.	School-level implementation file showing timetable coverage, responsible staff, learner feedback, incident-response pathways, resource availability, and follow-up actions. Document actions taken within one school term after safety, exclusion, or content-coverage concerns are identified.

Table 5 emphasises that implementation responsibility should not be displaced onto individual teachers alone. Policy, curriculum design, teacher education, and school management must operate together if sexuality education is to become routine, inclusive and accountable in classroom practice.

Implementation should follow a minimum accountability cycle: assign responsibility, fund the required inputs, verify curriculum coverage, collect protected learner feedback, review inequities by learner group, and document corrective action. Without this cycle, recommendations risk remaining aspirational and responsibility may again be displaced onto individual teachers.

The first implication is that policy must be accompanied by implementation infrastructure. South Africa should strengthen teacher education, school leadership, community engagement, disability-accessible materials and referral pathways, while protecting CSE from selective moral narrowing. Sweden should strengthen coordination across subjects, ensure that all teachers responsible for SCR receive appropriate training, audit classroom resources for silences, and monitor whether learner participation and marginalised-learner inclusion occur in practice (Andersson et al., 2020; Skolinspektionen, 2025).

The second implication is that teacher education should be redesigned around difficult knowledge. In both countries, teachers need preparation to teach consent, gender equality, pleasure, pornography, sexual orientation, gender identity, disability, intersex, asexuality, religion, culture, racism, migration, and digital sexual cultures. Training must also cover how to manage disclosure, distress, peer ridicule, discriminatory language, and classroom power relations (Andersson et al., 2020; Hildebrand, 2025; Junkala, 2023; Planting-Bergloo, 2023).

The third implication is that learner participation should become a formal quality indicator. Schools should be required to demonstrate how learners participate in planning, evaluation, and adaptation of sexuality education, while protecting vulnerable learners from forced disclosure or exposure. Anonymous learner feedback, marginalised-youth advisory mechanisms and regular inclusive-resource audits can help shift participation from tokenism to accountability (Koch and Beyers, 2023; Schindele et al., 2023; Skolinspektionen, 2018).

The fourth implication is that inclusion must be evaluated by content, pedagogy, and experience. It is not enough for a curriculum to mention LGBTQI+ learners, disability, or migration. Inclusive implementation should be assessed through whether learners encounter accurate, affirming, and non-tokenistic representations; whether teachers can respond to questions and disclosures; whether materials recognise diverse bodies and family forms; and whether students can access referral pathways without shame or discrimination (Hanass-Hancock et al., 2018; Junkala et al., 2022; Löfgren-Mårtenson, 2012).

### 4.5 Future research agenda

A more focused future research agenda is needed to move the field beyond general calls for more evidence. Table 6 identifies priority research questions, suitable methodological approaches and the evidence gaps that should be addressed next.

**Table 6:** Priority future research agenda for comparative sexuality-education implementation studies.

Priority research question or evidence gap	Recommended methodological approach	Expected contribution
How do implementation fragility and implementation drift appear at school level rather than only in policy and published evidence?	Comparative multi-site case studies using classroom observation, teacher interviews, learner focus groups, and school-document review.	Tests whether the proposed concepts explain real implementation variation across schools, subjects, and learner groups.
Which teacher-education components improve confidence and inclusive enactment of contested or sensitive sexuality-education content?	Mixed-methods intervention studies and longitudinal follow-up of pre-service and in-service teacher-development programmes.	Identifies which training models shift classroom practice rather than only improving teacher attitudes or self-reported confidence.
How do marginalised learners experience recognition, silence or harm in sexuality education?	Participatory qualitative research, anonymous learner surveys and intersectional analyses involving LGBTQI+ learners, learners with disabilities, migrants, rural learners, intersex, and asexual learners.	Generates learner-centred evidence for equity indicators and prevents policy analysis from substituting adult assumptions for student experience.
How do subject location and curriculum architecture shape what is taught and avoided?	Curriculum mapping, textbook/resource analysis and classroom ethnography across Life Orientation, Biology, Social Studies, and interdisciplinary settings.	Clarifies whether subject integration broadens content or disperses accountability and whether scripted materials standardise or narrow pedagogy.
What monitoring indicators best capture inclusive implementation?	Design-based research with education departments, inspectorates, schools, and youth advisory groups to co-develop and test implementation dashboards.	Produces practical accountability tools that assess teacher preparation, learner voice, inclusion, referral pathways, and classroom safety.
Which implementation strategies produce measurable improvements in learner knowledge, safety, inclusion, and help-seeking?	Quasi-experimental, stepped-wedge or cluster-randomised evaluations linked to implementation-process measures and disaggregated learner outcomes.	Addresses the current shortage of causal evidence and identifies scalable strategies rather than relying on descriptive recommendations.

Table 6 shows that future research should prioritise mechanism-testing, learner-centred evidence and implementation tools rather than only documenting whether sexuality education exists. Such research would strengthen both comparative theory-building and practical system improvement.

The immediate priority is comparative, school-level implementation research that links classroom observation with learner-reported outcomes. The second priority is prospective evaluation of pre-service and in-service teacher-education models. The third is the co-development and validation of equity-sensitive monitoring indicators. These priorities should use longitudinal mixed-methods designs, purposive inclusion of marginalised learners and cross-case sampling across rural, urban, mainstream, and adapted-school settings.

Future research should test the constructs of implementation fragility and implementation drift empirically. In South Africa, school-level studies should examine how CSE survives or narrows under community contestation, teacher beliefs, rural resource constraints, and school leadership variation. In Sweden, post-2022 and curriculum-reform follow-up studies should examine how SCR is enacted across subjects, how principals and teachers coordinate responsibility, and whether subject-specific relocation affects equity and quality.

Comparative research should also prioritise marginalised learners. This includes LGBTQI+ learners, learners with disabilities, intersex and asexual learners, migrant learners, rural learners and learners experiencing honour-related norms, stigma, or violence. Quantitative monitoring can identify inequities in perceived knowledge and access, while qualitative and participatory methods can show how classrooms produce recognition, silence or harm. Finally, implementation research should examine teacher education and professional development as causal mechanisms rather than as generic recommendations.

### 4.6 Strengths and limitations

This synthesis has four strengths. First, it documents search domains, Swedish and English search terms, eligibility rules and evidence weighting. Second, it expands the Swedish evidence base by incorporating policy, inspection, teacher-education mapping, doctoral theses, textbook studies, migrant-focused research, disability scholarship and learner-perspective evidence. Third, it explicitly addresses evidence imbalance and reflexivity regarding author-team contributions to the South African corpus. Fourth, it advances a theory-building contribution by distinguishing implementation fragility from implementation drift.

The limitations are also important. The synthesis is structured and critical rather than exhaustive; it does not claim to capture every relevant publication or to estimate effect sizes. Evidence types differ across countries: Sweden has more official inspection evidence, while South Africa has more context-specific empirical studies in the accessible corpus. Some Swedish sources are doctoral theses, reports or Swedish-language materials; although these are highly relevant, they are not identical in status to peer-reviewed journal articles. The synthesis also relies on published and publicly available evidence, which may underrepresent informal classroom practice, learner silence and schools without research visibility. Finally, implementation fragility and drift are analytical propositions that require further empirical testing. The absence of a formal risk-of-bias tool is appropriate to the heterogeneous corpus but limits direct comparability across source types; consequently, the review uses calibrated language and treats mechanism-level propositions as hypotheses for further testing rather than settled causal conclusions.

## 5 Conclusion

South Africa and Sweden demonstrate that formal policy commitment to sexuality education is a necessary but insufficient condition for equitable implementation. South Africa’s CSE architecture is vulnerable to implementation fragility when educator constraints, moral contestation, resource limitations and uneven school support prevent inclusive practice from becoming routine. Sweden’s SCR architecture is vulnerable to implementation drift when long-standing provision is assumed to be progressive while subject fragmentation, teacher-education variability, learner-participation gaps and unexamined silences weaken the equity promise of the curriculum.

Future research should therefore be organised around explicit questions of mechanism, equity and accountability: which implementation conditions protect sexuality education from fragility, which governance arrangements prevent drift, which forms of teacher education improve inclusive classroom enactment, and which learner-reported indicators best capture safety, relevance, and recognition. Priority should be given to multi-site longitudinal mixed-methods studies, direct classroom observation, participatory research with marginalised learners and prospective evaluation of teacher-development and monitoring interventions.

The comparative lesson is not that one system should copy the other. Rather, sexuality education systems should be governed as living implementation arrangements. They require coherent policy, trained and supported educators, meaningful learner participation, inclusive and regularly audited resources, disability- and migration-responsive pedagogy, referral pathways, and accountability measures that examine what learners actually experience. Moving from policy adoption to educational equity requires sustained attention to the classroom conditions under which all learners, especially marginalised learners, are recognised as rights-bearing subjects. For policymakers and education leaders, the immediate implication is to convert policy commitments into funded, assigned and auditable implementation responsibilities at district, school and classroom levels (Appendix A).

## Appendix A Search and source-verification protocol

**Table A1:** Search blocks and verification rules.

Search block	Example search terms	Primary source domains	Verification rule
South Africa policy	CSE; Life Orientation; Life Skills; HIV STI TB policy; scripted lesson plans	DBE, Gov.za, UNESCO Health and Education Resource Centre	Official DBE/Government source preferred; UNESCO records used for traceability.
South Africa implementation	teacher preparedness; sexuality diversity; learner perspectives; rural schools; disability; intersectoral collaboration	Publisher records, PubMed/PMC, Google Scholar, DOI records	Peer-reviewed empirical studies weighted for classroom implementation.
Sweden policy	sexuality consent relationships; sexualitet samtycke relationer; sex- och samlevnadsundervisning	Skolverket, Skolinspektionen, Public Health Agency of Sweden, Government Offices of Sweden	Official institutional records used for policy terminology and monitoring.
Sweden scholarship	sexualundervisning AND skola; ämnesintegrerad; normkritik; nyanlända; intellektuell funktionsnedsättning	Swepub, Libris, Libsearch, DiVA, publisher records, Google Scholar	Swedish-language records included where school-based implementation relevance was direct.
Cross-cutting theory	critical interpretive synthesis; policy triangle; implementation fidelity; institutional decoupling	Publisher records, PubMed/PMC, DOI records	Used to position concepts and method, not to infer country-specific implementation.
Citation verification	DOI, ISBN, official report title, institutional publication page	Crossref/DOI pages, publisher pages, institutional repositories	Sources without sufficient bibliographic traceability were not used as core evidence.

This table documents the search blocks and verification rules used to update and verify the evidence corpus in June 2026. The appendix is included to strengthen reproducibility while retaining the article as a critical policy and evidence synthesis rather than an exhaustive scoping review. This table should be read as an audit trail for reproducibility rather than as a claim of exhaustive systematic-review coverage. It strengthens transparency by showing how the search blocks, source domains and verification rules were aligned with the comparative policy-and-evidence synthesis design.
